# Trimethoprim-sulfamethoxazole Induced Pancytopenia: A Common Occurrence but A Rare Diagnosis

**DOI:** 10.7759/cureus.5071

**Published:** 2019-07-02

**Authors:** Priyanka Parajuli, Abdisamad M Ibrahim, Harris H Siddiqui, Odalys Estefania Lara Garcia, Manjari R Regmi

**Affiliations:** 1 Internal Medicine, Southern Illinois University School of Medicine, Springfield, USA; 2 Internal Medicine, Southern Illinois University School of Medicine, Springfield , USA

**Keywords:** pancytopenia, bactrim, trimethoprim sulfamethoxazole, tmp-smx, mrsa

## Abstract

Trimethoprim-sulfamethoxazole (TMP-SMX) is a bacteriostatic antimicrobial medication used for the treatment of a variety of infections and has many reported skin and hematologic side effects. Due to the easy availability and cost effectiveness, TMP-SMX is one of the medications commonly used for treatment of skin and soft tissue in patients with methicillin-resistant Staphylococcus aureus (MRSA) infection. One of the rare hematologic manifestations of TMP-SMX is pancytopenia, which is a reduction in all cell lines. In this case report, we are documenting a case of pancytopenia due to severe drug reaction to TMP-SMX in a 70-year-old female after two weeks of medication use. Upon initial stabilization she underwent a thorough workup and was subsequently diagnosed with severe drug-induced pancytopenia. Detailed history, early diagnosis, prompt discontinuation of the offending medication along with supportive care remain the mainstay of treatment in the management of TMP-SMX induced pancytopenia.

## Introduction

Pancytopenia is the reduction in all blood cell lines. A reduced production of the cell lines, intrinsic, or an increased destruction of the cell lines, extrinsic, can cause pancytopenia [1]. Pancytopenia can insidiously develop over a period. Alternatively, it can be of acute onset and progress quickly to disseminated intravascular coagulation, rapid hemolysis, and fulminant sepsis [2]. The differential diagnosis for a patient with pancytopenia is broad and the etiology can range from deficiency in nutrients such as folate and vitamin B12, use of antibiotics, and chemotherapeutic agents to transient viral marrow suppression and malignant bone marrow infiltration [3]. Drug-induced pancytopenia is an intrinsic cause of pancytopenia and is mostly considered a diagnosis of exclusion [1]. In this case report, we will discuss a case of a patient with drug-induced pancytopenia along with the differential diagnosis for pancytopenia of unknown origin and expected finding in each pathology.

## Case presentation

 A 70-year-old female presented to the ED with complaints of hematemesis, hemoptysis, melenic stools, and diffuse generalized body pain for four days. She denied any fevers, chills, lightheadedness, dizziness, chest pain, shortness of breath, or abdominal pain. There was no history of inflammatory bowel disease or gastric ulcers. On physical examination, she was in mild distress, hypotensive, tachycardic, and breathing room air. She had multiple ulcers throughout her hard palate, soft palate, and lips. Her abdomen was soft and nondistended with normal bowel sounds. After initial stabilization, she was admitted for further evaluation and management. CT scan of the chest, abdomen, and pelvis did not reveal any acute findings. Admission lab work is demonstrated below in Table 1.

**Table 1:** Laboratory data. Hgb, hemoglobin; MCV, mean corpuscular volume; WBC, white blood cell; Plt, platelet; ANC, absolute neutrophil count;  PT, prothrombin time; INR, international normalized ratio; PTT, partial thromboplastin time; AST, aspartate aminotransferase; ALT, alanine aminotransferase; LDH, lactate dehydrogenase; SPEP, serum protein electrophoresis; UPEP, urine protein electrophoresis; HIV, human immunodeficiency virus; CMV, cytomegalovirus; EBV, Epstein-Barr virus.

Lab parameter	Values	Reference range
Hgb	7.5 g/dL	12.0-16.0 gm/dL
MCV	77.0 fL	81-94 fL
WBC	0.7 K/CUMM	3.4-9.4 K/CUMM
Plt	9.0 K/CUMM	140-410 K/CUMM
ANC	0.1 K/CUMM	1.5-6.5 K/CUMM
Absolute reticulocyte count	0.1 %	0.5%-0.2 %
Absolute lymphocyte count	0.5 K/CUMM	0.9-3.0 K/CUMM
PT	14.1 s	11.9-14.9 s
INR	1.1	0.9-1.1
PTT	26.0 s	22.9-35.1 s
AST	16 IU/L	13-39 IU/L
ALT	11 IU/L	7-54 IU/L
LDH	112 IU/L	140-271 IU/L
Fibrinogen	756 mg/dL	200-400 mg/dL
Fibrin split products	20 uG/mL	0-9 uG/mL
D-dimer	1.18 mcg/mL	0.27-0.50 mcg/mL
Total bilirubin	0.7 mg/dL	0.3-1.0 mg/dL
Direct bilirubin	0.2 mg/dL	0.0-0.2 mg/dL
Vitamin B12	273 pg/mL	> = 200 pg/mL
Folate	7.5 ng/mL	> = 6.0 ng/mL
Peripheral smear	Marked pancytopenia. No blasts, spherocytes, or schistocytes	
Direct Coombs test	Negative	
SPEP and UPEP	No abnormal bands	
Hepatitis B, C, HIV, CMV, and EBV serologies	Negative	

Two weeks prior to admission her hemoglobin was slightly decreased at 11.0 g/dL. Her WBC count and platelet count were both within normal limits. The only new medication patient took from the time of onset of her symptoms leading to her admission was TMP-SMX for right lower extremity cellulitis. Over the course of her admission, her TMP-SMX was stopped and she was transfused two units of platelets and three units of packed RBCs. She was also treated with recombinant human granulocyte colony-stimulating factor (rhG-CSF). At a follow-up visit two months later, the patient’s complete blood count (CBC) had normalized.

## Discussion

According to the Infectious Diseases Society of America (IDSA) guidelines, routine treatment for community-acquired MRSA in patients presenting with purulent cellulitis is TMP-SMX [4]. It is a sulfonamide drug and is a combination of two antimicrobial agents that work sequentially to inhibit enzyme systems involved in the bacterial synthesis of tetrahydrofolic acid (THF). Reduced availability of THF inhibits thymidine synthesis, which in turn inhibits DNA synthesis [5-6]. The more common adverse reactions to TMP-SMX involve gastrointestinal tract (nausea, vomiting) and skin (rash and pruritus) [7]. However, a few life-threatening side effects such as pancytopenia, Steven-Johnson syndrome, hepatitis, and renal tubular acidosis have also been reported [7]. Blood dyscrasias as a result of TMP-SMX use was reported at 1 in 18,000 in a Swedish population study [8]. A population-based study in Seattle, Washington area concluded low hospitalization rate for blood disorder secondary to use of TMP-SMX [9].

Pancytopenia is a known side effect of TMP-SMX; however, it has not attracted much attention in the past few years and it is reflected by the sparsity of publications. The likely causes of pancytopenia are influenced by geography, socioeconomic conditions, and endemic illnesses [2]. The vast majority of pancytopenia in adults is caused by acquired disorders, such as infections, nutritional deficiencies, medications, and cancers [3].

**Figure 1:**
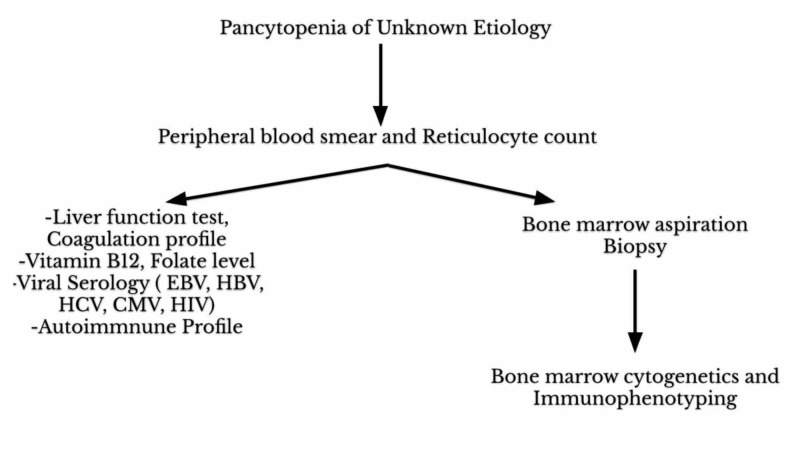
Diagnostic approach for patients presenting with pancytopenia of unknown etiology. HIV, human immunodeficiency virus; CMV, cytomegalovirus; EBV, Epstein-Barr virus; HBV, hepatitis B virus; HCV, hepatitis C virus.

**Figure 2:**
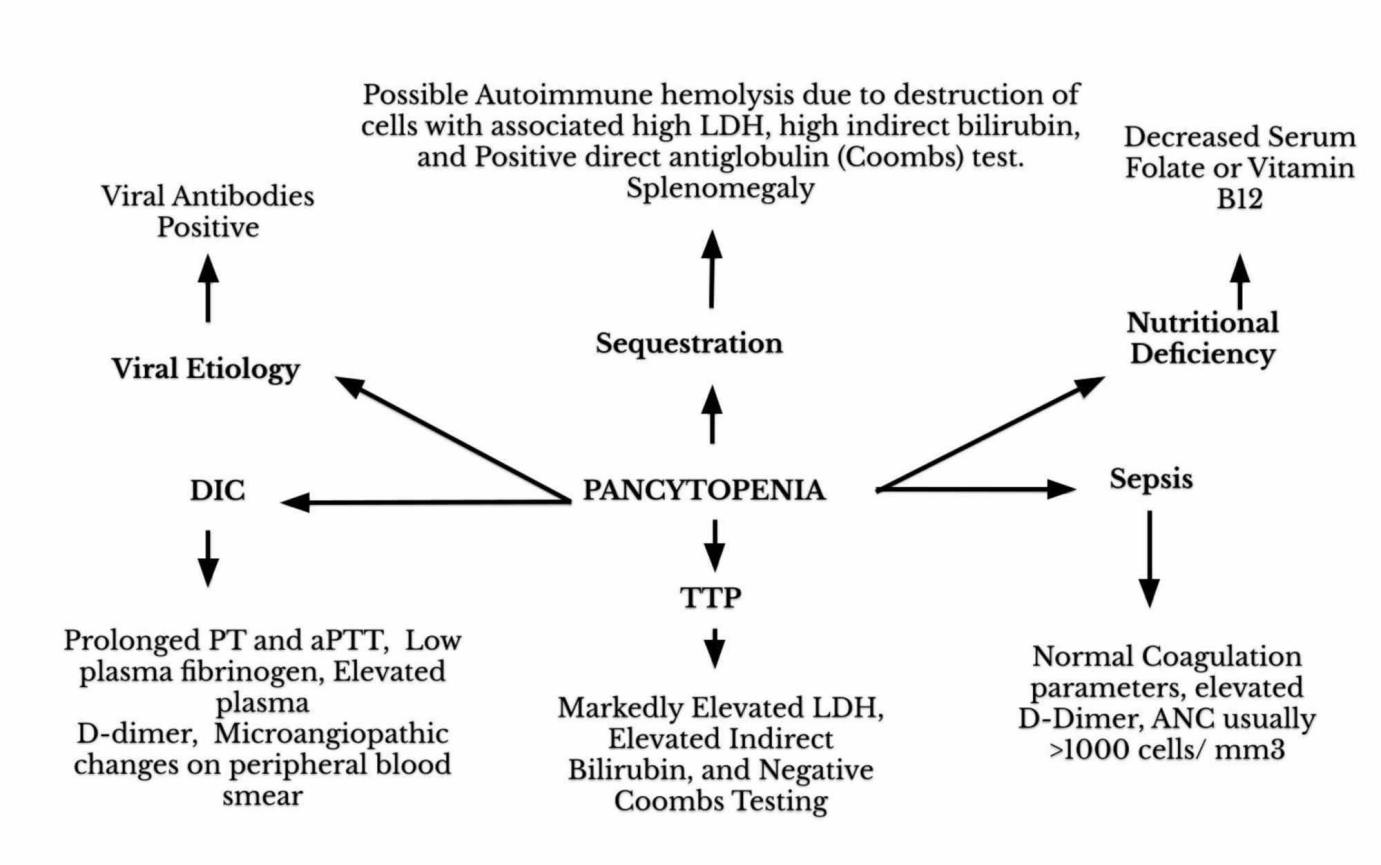
Differential diagnoses and expected findings in pancytopenia of various etiologies. LDH, lactate dehydrogenase; TTP, thrombotic thrombocytopenic purpura; aPTT, activated partial thromboplastin time; ANC, absolute neutrophil count.

In our report, the patient was started on TMP-SMX to cover MRSA for purulent cellulitis of her right lower leg. Initially, the patient tolerated TMP-SMX well with no side effects. However, after two weeks of treatment, she presented to the ED with hematemesis and coffee-ground emesis. She was diagnosed with drug-induced pancytopenia after ruling out disseminated intravascular coagulation (DIC), thrombotic thrombocytopenic purpura (TTP), nutritional deficiency, viral etiology, possible autoimmune disorder, and sepsis. In the following figures, we have summarized the diagnostic approach for patients presenting with pancytopenia of unknown etiology and unique features of each differential diagnosis as Figures 1-2, respectively.

 

## Conclusions

The differential diagnoses in a patient with acute onset pancytopenia are very comprehensive. It is important to understand that TMP-SMX can cause an idiosyncratic allergic reaction leading to pancytopenia. However, due to inadequate reports regarding such reaction drug-related pancytopenia is not a leading differential diagnosis and can affect the management of patients. Therefore, detailed history along with thorough physical examination and judicious laboratory investigation becomes extremely essential in investigating the cause of pancytopenia to initiate appropriate treatment.

## References

[1] Kelm DJ, Torres KM, Sohail MR (2012). 46-year-old man with fevers, chills, and pancytopenia. Mayo Clin Proc.

[2] Sharma R, Nalepa G (2016 ). Evaluation and management of chronic pancytopenia. Pediatr Rev.

[3] Crews J, Dawkins R (2016). Acute-onset pancytopenia in a previously healthy teenager. J Paediatr Neonatal Dis.

[4] Liu C, Bayer A, Cosgrove SE (2011). Clinical practice guidelines by the Infectious Diseases Society of America for the treatment of methicillin-resistant Staphylococcus aureus infections in adults and children. Infect Dis.

[5] Kalkut G (2009). Sulfonamides and trimethoprim. Cancer Invest.

[6] Gleckman R, Blagg N, Joubert DW (1981). Trimethoprim: mechanisms of action, antimicrobial activity, bacterial resistance, pharmacokinetics, adverse reactions, and therapeutic indications. Pharmacotherapy.

[7] Cunha BA (2001 ). Antibiotic side effects. Med Clin North Am.

[8] Keisu M, Wiholm BE, Palmblad J (1990). Trimethoprim‐sulphamethoxazole‐associated blood dyscrasias. Ten years' experience of the Swedish spontaneous reporting system. J Intern Med.

[9] Myers MW, Jick H (1997). Hospitalization for serious blood and skin disorders following co-trimoxazole. Br J Clin Pharmacol.

